# Surgical management of intraoperatively diagnosed portal annular pancreas: Two case reports

**DOI:** 10.1097/MD.0000000000028204

**Published:** 2021-12-17

**Authors:** Nobutaka Abe, Sang-Woong Lee, Tetsunosuke Shimizu, Mitsuhiro Asakuma, Kohei Taniguchi, Atsushi Tomioka, Fumitoshi Hirokawa, Kazuhisa Uchiyama

**Affiliations:** a Department of General and Gastroenterological Surgery, Osaka Medical and Pharmaceutical University, Daigaku-machi, Takatsuki, Osaka, Japan; b Translational Research Program, Osaka Medical and Pharmaceutical University, Daigaku-machi, Takatsuki, Osaka, Japan.

**Keywords:** case report, distal pancreatectomy, pancreas, pancreaticoduodenectomy, portal annular pancreas

## Abstract

**Rationale:**

Portal annular pancreas (PAP) is a rare pancreatic anomaly characterized by portal vein encasement in the pancreatic parenchyma. Due to its rarity, PAP may often be missed on preoperative computed tomography (CT) review, and surgeons may face challenges in dealing with an unexpected intraoperative encounter with PAP. We documented 2 such intraoperatively diagnosed cases and illustrated their surgical management.

**Patients’ concerns:**

In case 1, a 70-year-old man was found to have a 15-mm mass in the pancreatic body and dilatation of the peripheral main pancreatic duct on enhanced CT. Case 2 involved a 46-year-old woman with a history of familial adenomatous polyposis, and rectal cancer with a mass in the duodenal papilla.

**Diagnoses:**

The patient in case 1 was diagnosed with resectable pancreatic cancer. In case 2, the patient was diagnosed with duodenal papillary carcinoma.

**Interventions:**

In case 1, the patient underwent distal pancreatectomy with lymph node dissection. In case 2, the patient underwent pancreaticoduodenectomy. Intraoperatively, PAP was observed in both cases. In case 1, after the usual transection at the right border of the portal vein, an additional dissection was performed on the dorsal pancreas using a powered linear stapler. In case 2, an additional section was made in the pancreatic body caudal to the cricoid pancreatic junction so that the pancreatic cross-section was oriented in 1 plane.

**Outcomes:**

The patient in case 1 was discharged without complications. In case 2, although the patient had a grade-B pancreatic fistula (International Study Group of Pancreatic Fistula Classification), the patient recovered conservatively and was discharged without significant complications. In both cases, a retrospective review identified PAP in patients’ preoperative CT images.

**Lessons:**

Both cases required ingenuity during pancreatectomy. Awareness about PAP and its management will enable surgeons to prepare for unexpected encounters with the condition. Moreover, surgeons (especially pancreatic surgeons) should consider the possibility of PAP while managing pancreatic anomalies to make appropriate treatment decisions.

## 1 Introduction

There are 2 types of annular pancreas: portal annular pancreas (PAP) and duodenal annular pancreas. PAP is a rare pancreatic anomaly characterized by the encasement of either the portal vein (PV) or superior mesenteric vein in the pancreatic parenchyma. PAP was first reported by Sugiura et al^[1]^ in 1987. It is usually asymptomatic and discovered incidentally during surgery. In 1 study, it was observed in 1.1% to 2.5% of patients who randomly underwent computed tomography (CT).^[2]^ Herein, we report 2 cases of PAP that were discovered during distal pancreatectomy (DP) and pancreaticoduodenectomy (PD) to illustrate its management when encountered unexpectedly.

## 2 Case reports

### 2.1 Case 1

**Figure 1:**
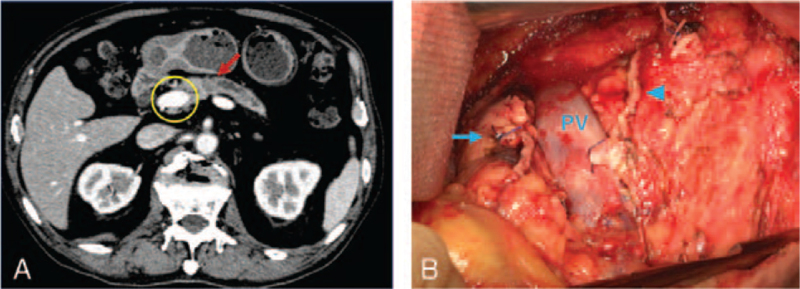
Computed tomography and intraoperative findings in case 1. (A) The image shows a 15-mm mass in the pancreatic body (red arrow), dilatation of the caudal end of the main pancreatic duct, and the portal annular pancreas (yellow circle). (B) Following pancreatic transection, the ventral (blue arrow) and dorsal (blue arrowhead) margins of the portal annular pancreas can be observed. PV = portal vein.

A 70-year-old man with a high carbohydrate antigen 19-9 level was diagnosed with resectable pancreatic cancer. Enhanced CT revealed a 15-mm mass in the pancreatic body and dilatation of the peripheral main pancreatic duct (Fig. 1A). The patient underwent DP with lymph node dissection. DP was performed by transecting the pancreas at the right border of the PV with a powered linear stapler. Additional pancreatic tissue was observed on the dorsal aspect of the PV (Fig. 1B), and the patient was diagnosed with PAP (type II). After the diagnosis, additional pancreatic resection was conducted using a powered linear stapler. The total operative time was 527 minutes, and the estimated blood loss was 730 mL. Based on the Union for International Cancer Control TNM staging, the pathological diagnosis was T3N0M0, stage IIA. In addition, the pathological margin was negative. The patient had no complications and was discharged on postoperative day 23. PAP was detected on preoperative CT images during a retrospective review (Fig. 1A).

### 2.2 Case 2

**Figure 2:**
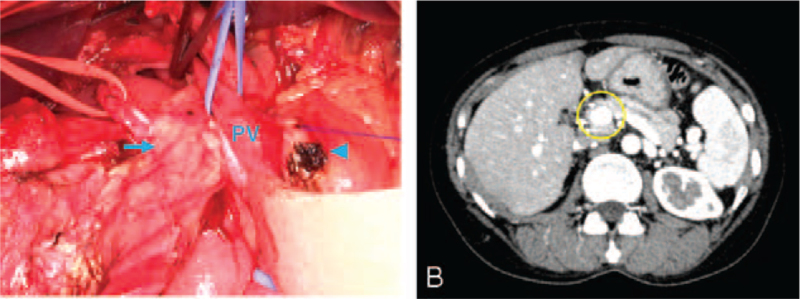
Computed tomography and intraoperative findings in case 2. (A) Following pancreatic transection, the dorsal (blue arrow) and ventral (blue arrowhead) margins of the portal annular pancreas can be observed. (B) The image shows the portal annular pancreas (yellow circle). PV = portal vein.

A 46-year-old woman with a history of familial adenomatous polyposis and rectal cancer underwent PD with lymph node dissection for a newly diagnosed duodenal papillary carcinoma. Pancreatectomy was performed by transecting the pancreas superior to the PV. The pancreatic parenchyma was subsequently identified on the dorsal side of the PV (Fig. 2A) and transected, resulting in the exposure of a section (2 mm in length) of the main pancreatic duct. The remaining pancreas was mobilized caudally. An additional section was made in the pancreatic body caudal to the cricoid pancreatic junction so that the pancreatic cross-section was oriented in 1 plane. The diagnosis was PAP (type II). The total operative time was 439 minutes, and the estimated blood loss was 250 mL. Based on the Union for International Cancer Control TNM staging, the pathological diagnosis was pT1aN0M0, stage IA. In addition, the pathological margin was negative.

Although the patient had a grade-B pancreatic fistula (International Study Group of Pancreatic Fistula Classification) that was diagnosed on postoperative day 3, the patient recovered after conservative treatment, which is continuous drainage until 2 days before discharge, and was discharged on postoperative day 18. We retrospectively reviewed the patients’ preoperative CT images and identified the PAP (Fig. 2B).

## 3 Discussion

In this case report, we describe 2 cases of PAP that were diagnosed intraoperatively. According to Joseph et al,^[3]^ PAP can be classified into 5 types and both of our cases were type-II PAP. Pancreatic transection of PAP during both DP and PD is often difficult, particularly with type-II PAP; the main and accessory pancreatic ducts run dorsal and ventral to the PV,^[3]^ as was the case in our patients.

When we performed DP with a powered linear stapler, we proposed that this procedure be performed with an intestinal clamp using the slow compression method, which has been reported to be useful in all DP surgeries with any PAP type.^[4]^ Briefly, first, the pancreas was compressed for 3 minutes using the intestinal clamp with 3 clicks out of 9 clicks, followed by an additional 3 clicks for 3 minutes. Finally, the last 3 clicks were used for 3 minutes. After removing the intestinal clamp, the compression line was visible. After caudally shifting the transection line on the compression site, the stapler was fired for approximately 1 minute. We believe that this technique can also be safely applied to the slow compression method to decrease the risk of postoperative complications, especially pancreatic fistula formation.

Comparatively, during PD for types I and III PAP, the pancreatic transection line is often located along the left side of the superior mesenteric vein. Attention should be paid to type-II PAP since it is vital to set the transection plane peripherally to create a single pancreatic ductal plane. In case 2, we transected the pancreas at a line superior to the superior mesenteric artery without first confirming the location of the ductal conjunction. After the transection, we confirmed the pancreatic parenchyma on the dorsal side of the PV. This parenchyma was continuous from the dorsal side of the common hepatic artery to the celiac artery. We resected the remnant pancreas using an electric knife. This procedure was risky and could have resulted in serious postoperative complications, such as pancreatic fistula formation. Matsumoto et al^[5]^ reported that intraoperative pancreatography is a valuable tool for identifying the distal end of the duct junction, which serves as a reference point for making the transection line. Narita et al^[6]^ achieved an uneventful postoperative course by performing telescopic pancreaticogastrostomy in a case with 2 main pancreatic ducts. Furthermore, following pancreatectomy of the dorsal side of the pancreas, Sugiura et al^[1]^ anastomosed the ventral side of the pancreas to a section of the intestine. Several methods for anastomosis have since been reported, none of which has proven superiority to date. The current consensus is to perform an anastomosis regardless of the number of pancreatic ducts. We believe that pancreatectomy in PAP is also associated with an increased intraoperative bleeding risk due to the proximity of the PV and that extra caution while working in the area around the PV may prolong the operative time.

A major limitation of this report is that a preoperative diagnosis was not made. To resolve this, surgeons (especially pancreatic surgeons) should have a sound knowledge of PAP, keep in mind the possibility of the existence of PAP, consult with a radiologist when in doubt, and be knowledgeable about the anatomy of structures surrounding the PV. According to a previous report, CT is sufficient to diagnose the presence of PAP.^[7]^ Thus, CT is the optimal imaging modality. When the presence of PAP can be pointed out on preoperative CT, additional tests such as Magnetic Resonance Cholangio Pancreatgraphy can be used to examine the pancreatic ducts before surgery. Therefore, pancreatic surgeons should be aware of PAP in preoperative situations.

In conclusion, PAP is a rare pancreatic anomaly. If the presence of PAP is confirmed preoperatively, the location of the pancreatic transection line should be planned before surgery. However, if it is discovered intraoperatively, awareness of PAP and its management will enable surgeons to respond in an appropriate manner. In short, the 2 times of resection using the slow compression method in the case of DP is safe. The safe method is to transect the remnant pancreas on the dorsal side of PV, thereby confirming the number of main pancreatic ducts in the case of PD. Hence, pancreatic surgeons should always consider the possibility of PAP to make an appropriate treatment decision.

## Acknowledgments

The authors are grateful for the cooperation of all the staff engaged in patient management. We would like to thank Editage (www.editage.com) for English language editing.

## Author contributions

NA, KT, and TS designed the study. MA, AT, FH, and KU performed the surgery and provided preoperative care. NA drafted the manuscript. MA, AT, FH, and KU analyzed and interpreted the data. KT, TS, and SWL reviewed and revised the manuscript. KU supervised the study. All authors read and approved the final manuscript.

**Supervision:** Sang-Woong Lee.

**Writing – original draft:** Nobutaka Abe.

**Writing – review & editing:** Tetsunosuke Shimizu, Mitsuhiro Asakuma, Kohei Taniguchi, Atsushi Tomioka, Fumitoshi Hirokawa, Kazuhisa Uchiyama.

## References

[1] Sugiura Y Shima S Yonekawa H Yoshizumi T Ohtsuka H Ogata T. The hypertrophic uncinate process of the pancreas wrapping the superior mesenteric vein and artery-a case report. Jpn J Surg 1987;17:182–5. 3626212 10.1007/BF02470596

[2] Karasaki H Mizukami Y Ishizaki A. Portal annular pancreas, a notable pancreatic malformation: frequency, morphology, and implications for pancreatic surgery. Surgery 2009;146:515–8. 19715809 10.1016/j.surg.2009.03.018

[3] Joseph P Raju RS Vyas FL Eapen A Sitaram V. Portal annular pancreas. A rare variant and a new classification. JOP 2010;11:453–5. 20818114

[4] Shimizu T Miyamato Y Asakuma M. A novel technique of slow compression using the intestinal clamp for distal pancreatectomy. JOP 2016;17:209–12.

[5] Matsumoto I Shinzeki M Fukumoto T Ku Y. An extremely rare portal annular pancreas for pancreaticoduodenectomy with a special note on the pancreatic duct management in the dorsal pancreas. Surgery 2013;153:434–6. 22000830 10.1016/j.surg.2011.08.017

[6] Narita M Hata H Ikai I. Portal annular pancreas: an unusual pancreatic anomaly. J Visc Surg 2016;153:153–5. 26867987 10.1016/j.jviscsurg.2015.12.005

[7] Harnoss JM Harnoss JC Diener MK. Portal annular pancreas: a systematic review of a clinical challenge. Pancreas 2014;43:981–6. 25207658 10.1097/MPA.0000000000000186 PMC4175015

